# An Early Pandemic Analysis of SARS-CoV-2 Population Structure and Dynamics in Arizona

**DOI:** 10.1128/mBio.02107-20

**Published:** 2020-09-04

**Authors:** Jason T. Ladner, Brendan B. Larsen, Jolene R. Bowers, Crystal M. Hepp, Evan Bolyen, Megan Folkerts, Krystal Sheridan, Ashlyn Pfeiffer, Hayley Yaglom, Darrin Lemmer, Jason W. Sahl, Emily A. Kaelin, Rabia Maqsood, Nicholas A. Bokulich, Grace Quirk, Thomas D. Watts, Kenneth K. Komatsu, Victor Waddell, Efrem S. Lim, J. Gregory Caporaso, David M. Engelthaler, Michael Worobey, Paul Keim

**Affiliations:** a Pathogen and Microbiome Institute, Northern Arizona University, Flagstaff, Arizona, USA; b Department of Ecology and Evolutionary Biology, University of Arizona, Tucson, Arizona, USA; c Pathogen and Microbiome Division, Translational Genomics Research Institute, Flagstaff, Arizona, USA; d School of Informatics, Computing, and Cyber Systems, Northern Arizona University, Flagstaff, Arizona, USA; e Center for Applied Microbiome Science, Pathogen and Microbiome Institute, Northern Arizona University, Flagstaff, Arizona, USA; f Arizona Department of Health Services, Phoenix, Arizona, USA; g Bureau of Laboratory Services, Arizona Department of Health Services, Phoenix, Arizona, USA; h School of Life Sciences, Arizona State University, Tempe, Arizona, USA; i Center for Fundamental and Applied Microbiomics, Biodesign Institute, Tempe, Arizona, USA; j Department of Biological Sciences, Northern Arizona University, Flagstaff, Arizona, USA; University of Maryland, School of Medicine

**Keywords:** Arizona, COVID-19, genome analysis, molecular clock, phylogenetic analysis

## Abstract

In December of 2019, a novel coronavirus, SARS-CoV-2, emerged in the city of Wuhan, China, causing severe morbidity and mortality. Since then, the virus has swept across the globe, causing millions of confirmed infections and hundreds of thousands of deaths. To better understand the nature of the pandemic and the introduction and spread of the virus in Arizona, we sequenced viral genomes from clinical samples tested at the TGen North Clinical Laboratory, the Arizona Department of Health Services, and those collected as part of community surveillance projects at Arizona State University and the University of Arizona. Phylogenetic analysis of 84 genomes from across Arizona revealed a minimum of 11 distinct introductions inferred to have occurred during February and March. We show that >80% of our sequences descend from strains that were initially circulating widely in Europe but have since dominated the outbreak in the United States. In addition, we show that the first reported case of community transmission in Arizona descended from the Washington state outbreak that was discovered in late February. Notably, none of the observed transmission clusters are epidemiologically linked to the original travel-related case in the state, suggesting successful early isolation and quarantine. Finally, we use molecular clock analyses to demonstrate a lack of identifiable, widespread cryptic transmission in Arizona prior to the middle of February 2020.

## Abstract (precis)

As the COVID-19 pandemic swept across the United States, there was great differential impact on local and regional communities. One of the earliest and hardest hit regions was in New York, while at the same time Arizona (for example) had low incidence. That situation has changed dramatically, with Arizona now having the highest rate of disease increase in the country. Understanding the roots of the pandemic during the initial months is essential as the pandemic continues and reaches new heights. Genomic analysis and phylogenetic modeling of SARS-COV-2 in Arizona can help to reconstruct population composition and predict the earliest undetected introductions. This foundational work represents the basis for future analysis and understanding as the pandemic continues.

## INTRODUCTION

In late 2019, a novel positive-sense RNA virus, family *Coronaviridae*, genus *Betacoronavirus*, and subgenus *Sarbecovirus*, emerged in the human population due to cross-species transmission from an unknown host (1, 2). The virus, SARS-CoV-2, began widespread circulation in the Chinese city of Wuhan in late December of 2019, with the first cases in other countries detected around mid-January 2020 (3, 4).

Arizona’s first confirmed case of COVID-19, the disease caused by SARS-CoV-2, was detected in late January 2020 in a student attending Arizona State University who had traveled to China (5). Extensive contact tracing and isolation led to zero additional reported cases stemming from this original case (AZ1). There were no additional cases reported in Arizona until 3 March, when a traveler returned from Europe and tested positive (6). On 6 March, the first case of community transmission in Arizona was announced (7). On 26 March, the Arizona Department of Health Services (AZDHS) updated the status of community transmission to “widespread.” At the time of writing this paper, there were ∼91,860 confirmed positive cases in Arizona, and more than 1,780 deaths (8), where the number of new cases and deaths per day increased by a factor of 10 and 4 over the previous 2 months, respectively (9). The Navajo Nation, located mostly in northeastern Arizona (but also Utah, Colorado, and New Mexico) had among the highest number of cases per capita in the United States: approximately 4,390 confirmed cases per 100,000 individuals (10). This rate was disproportionately high relative to the rest of Arizona (approximately 1,280 confirmed cases per 100,000 individuals), and about 200% of the per capita cases seen in the hardest-hit regions of the United States (e.g., New York [11]).

Given the scale of the pandemic, there is an urgent need to understand patterns of SARS-CoV-2 spread, including the relative roles of local transmission versus repeated travel-associated introductions, and the accumulation and spread of mutations that could affect the function of the virus, interfere with testing, or have antigenic effects that might impact vaccine efforts. Viral genome sequencing has emerged as a key tool for addressing these questions. In order to assist with both local and global efforts to track the spread and evolution of this virus, we began intensive sequencing of viral genomes from across Arizona and deposition of these sequences in the GISAID database, which makes them accessible to the research community for downstream analyses, notably including real-time pathogen tracking through Nextstrain (12).

To better understand the evolution of the virus within the state of Arizona, we compared our sequences with publicly available SARS-CoV-2 genomes from across the world in a phylogenetic framework, and we report here our preliminary findings. Specifically, we sought to answer three key questions regarding the circulation of the virus in Arizona. First, did the initial case of COVID-19 in Arizona lead to cryptic community transmission that helped to fuel the ongoing epidemic? (13) Second, how many independent introductions contributed to the outbreak in Arizona and what was the approximate timing of each event? Third, are there any unique mutations present in Arizona sequences that could have potential phenotypic effects or could interfere with diagnostic detection?

## RESULTS AND DISCUSSION

As of 5 April 2020, we have sequenced and assembled a total of 79 nearly complete SARS-CoV-2 genomes obtained from patients across Arizona. These genomes were sequenced from nasopharyngeal swabs that were collected over a 28-day period from 5 March to 2 April 2020. This represents a sequencing effort of 4.9% of all reported cases in Arizona as of 2 April. This data set includes at least one genome from 11 of the 15 Arizona counties (see Table S3 in the supplemental material). We also included five SARS-CoV-2 genomes from Arizona that were sequenced by the Centers for Disease Control and Prevention (CDC), for a total of 84 Arizona sequences. The CDC sequences include the genome generated from the first documented case in Arizona (AZ1, collected 22 January 2020), as well as four cases that occurred in early March (see Table S1).

10.1128/mBio.02107-20.4 TABLE S1 Metadata for all SARS-CoV-2 genome sequences used in reported analyses. Download Table S1, XLSX file, 0.1 MB. Copyright © 2020 Ladner et al. 2020 Ladner et al. This content is distributed under the terms of the Creative Commons Attribution 4.0 International license.

10.1128/mBio.02107-20.5 TABLE S2 Non-synonymous substitutions observed in SARS-CoV-2 genomes sampled in Arizona. Download Table S2, XLSX file, 0.1 MB. Copyright © 2020 Ladner et al. 2020 Ladner et al. This content is distributed under the terms of the Creative Commons Attribution 4.0 International license.

10.1128/mBio.02107-20.6 TABLE S3 Genome representation from each Arizona county. This does not include all genomes because some are from unknown counties. Download Table S3, PDF file, 0.1 MB. Copyright © 2020 Ladner et al. 2020 Ladner et al. This content is distributed under the terms of the Creative Commons Attribution 4.0 International license.

### The initial Arizona case did not lead to sustained local transmission.

The first case of COVID-19 in Arizona (AZ1) was documented in late January 2020, and contact tracing suggested that this initial case did not result in additional symptomatic infections within the state (5). We sought to independently verify this conclusion using Bayesian and maximum-likelihood phylogenetic analyses to compare all 84 Arizona genomes to a representative subset of the SARS-CoV-2 genomes generated from around the world. The genomes used in this analysis were selected using a novel bioinformatics pipeline (see Materials and Methods), which subsampled genomes uploaded to GISAID to reduce the size of the data set while representing the temporal, spatial, and genetic diversity of the full data set. This resulted in a set of 388 SARS-CoV-2 genome sequences, including 84 Arizona genomes and 304 additional representatives (see Table S1). A reduced version of this data set, including 376 genomes with complete date information, was used in the Bayesian analysis.

**FIG 1:**
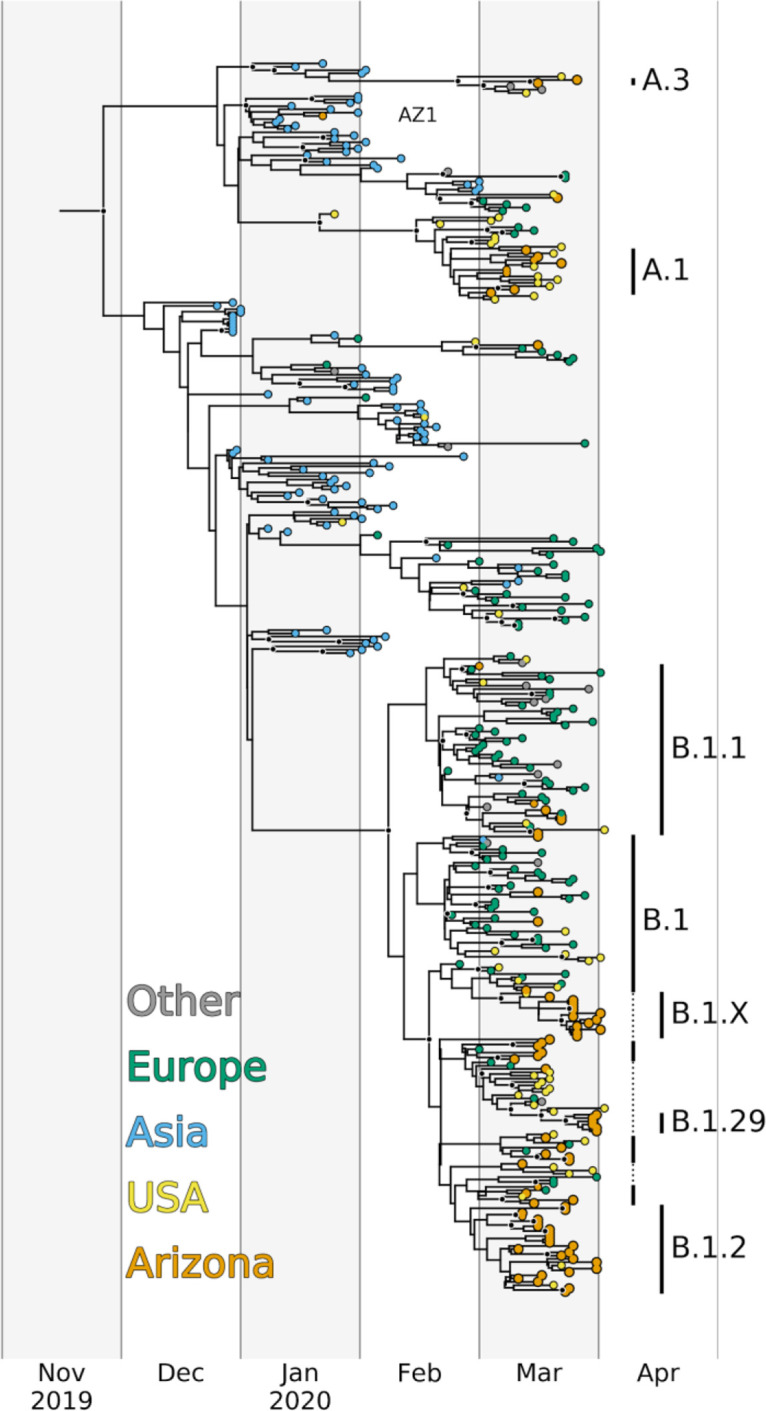
Bayesian maximum clade credibility time-calibrated phylogeny inferred from 376 SARS-CoV-2 genomes, including 84 from Arizona and 292 representatives from around the world. Tips are colored by origin of sequence, and major lineages assigned by Pangolin (https://github.com/cov-lineages/pangolin) with more than two sequence representatives in Arizona are indicated by vertical bars. B.1.X is a well-supported sublineage of B.1 that has not been named by Pangolin. All nodes with posterior probabilities >0.9 are colored black. The tree was visualized with a custom Python script that utilized the software package BALTIC (https://github.com/evogytis/baltic).

Our phylogenetic analyses (Fig. 1; see also Fig. S1) indicated that the genome from AZ1 belonged to lineage A (all lineage names are according to the Pangolin nomenclature [14]). Although 11/83 (13%) of the remaining Arizona genomes also clustered in lineage A, these genomes belonged to distinct sublineages (A.1, A.2, and A.3), and the AZ1 genome contained one derived substitution (C to T at nucleotide position 29,031) that we did not observe in any other Arizona sequences. This substitution was, however, shared with 12 other genomes included in our analysis, all of which were sampled in China or Japan. This is consistent with infection of AZ1 occurring during documented travel to China (5).

10.1128/mBio.02107-20.2 FIG S1 Maximum-likelihood phylogeny inferred from 388 SARS-CoV-2 genomes, including 84 from Arizona and 304 representatives from around the world. Tips are colored by origin of sequence, and major lineages assigned by Pangolin, with more than two sequence representatives from Arizona, are labeled and indicated by black vertical bars. (Note that the vertical bars do not necessarily cover all genomes from a given Pangolin lineage, only those from Arizona.) B.1.X is a well-supported sublineage of B.1 that has not been named by Pangolin. Genomes from Arizona are labeled with the associated GISAID accession number and Arizona County of origin, when available. The tree was visualized with a custom Python script that utilized the software package BALTIC (https://github.com/evogytis/baltic). Download FIG S1, PDF file, 0.6 MB. Copyright © 2020 Ladner et al. 2020 Ladner et al. This content is distributed under the terms of the Creative Commons Attribution 4.0 International license.

Although our analyses cannot completely rule out the possibility of a cryptic transmission chain originating from this initial case, it is clear that the first documented introduction of SARS-CoV-2 to Arizona did not play a substantial role in fueling the ongoing epidemic. Rather, most Arizona cases are linked to later introduction events (see below). These results demonstrate the power of public health contact tracing and self-isolation following a positive test for stemming the tide of infections moving forward.

### Multiple introductions have contributed to transmission in Arizona.

**FIG 2:**
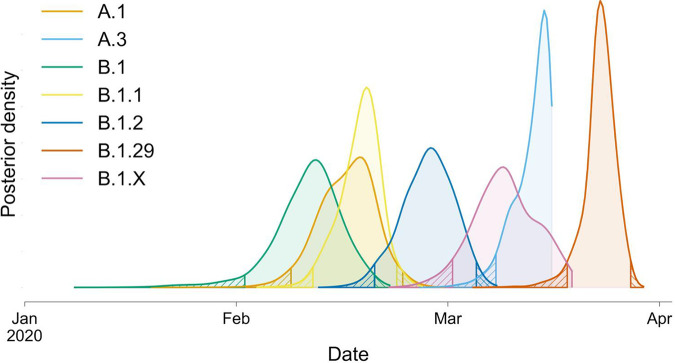
Posterior density estimates of TMRCAs for Arizona genomes that belong to seven major lineages/sublineages. Posterior density estimates were parsed from 12,001 trees sampled from four independent MCMC chains, following burn-in removal. Hatch marks indicate regions outside the 95% HPD. The samples included in each lineage can be seen in Fig. 1.

**FIG 3:**
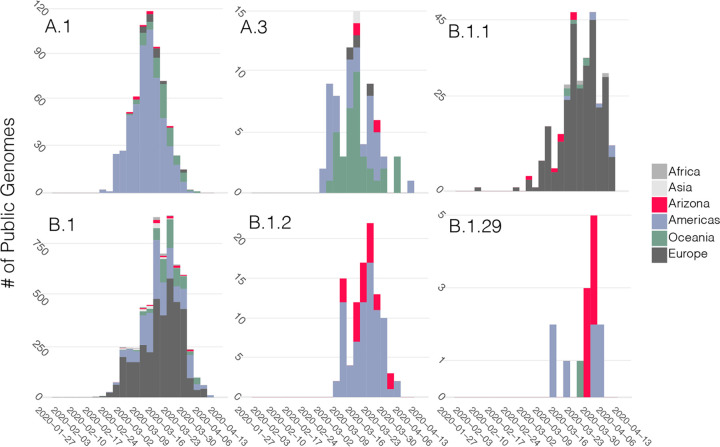
Sequence database representation through time for each of the six major named lineages or sublineages observed in Arizona. Stacked bars are colored according to location. To estimate A.1, B.1, and A.3, nested sublineages were collapsed to calculate the frequencies for the broader clades. Lineages were assigned using Pangolin (37) for all sequences uploaded to GISAID as of 16 April 2020.

Our phylogenetic analyses indicated that multiple distinct SARS-CoV-2 lineages cocirculated in AZ during March 2020. We detected 11 distinct lineages and/or sublineages in Arizona between early March and early April (Table 1), including one that was unnamed by Pangolin, but contained 13 Arizona genomes and 1 genome from Connecticut and was well-supported in both of our phylogenetic analyses (B.1.X). None of these lineages were unique to Arizona; they have all been documented in other parts of the United States and, in most cases, also in multiple countries around the world (Fig. 2 and 3; see also Fig. S2). In fact, the geographic ubiquity of the major SARS-CoV-2 lineages, along with the common observation of identical virus genomes sampled in multiple U.S. states, as well as other countries and continents, clearly demonstrates how frequently this virus has been moved among locales. Given the timing and size of the Arizona outbreak during the period of investigation, relative to outbreaks in other locations (Fig. 3), we argue it is unlikely that any of these 11 lineages arose within Arizona. Therefore, the number of observed distinct lineages (12, including AZ1) represents a conservative estimate for the number of independent introductions of SARS-CoV-2 into Arizona.

**TABLE 1:** Information on sequence number, timing, and location of each of the lineages detected in Arizona

Lineage	No. of sequences	Date collected in AZ	County(ies)
A	1	1/22/20	Maricopa
A.1	8	3/5/2020 to 3/23/2020	Graham, Maricopa, Mohave, Pima, Pinal
A.2	1	3/22/20	Cochise
A.3	2	3/17/20 to 3/27/20	Coconino
B.1	19	3/11/20 to 4/2/20	Coconino, Maricopa, Navajo, Pima, Pinal, Yuma
B.1.1	6	3/2/20 to 3/23/20	Coconino, Maricopa, Pinal
B.1.X	13	3/20/20 to 4/2/20	Maricopa
B.1.2	25	3/12/20 to 4/1/20	Coconino, La Paz, Maricopa, Navajo, Pinal, Yavapai
B.1.21	1	3/13/20	Pima
B.1.29	6	3/13/20 to 4/1/20	Maricopa
B.1.3	1	3/19/20	Coconino
B.2	1	3/17/20	Coconino

10.1128/mBio.02107-20.3 FIG S2 Sequence database representation through time for the four introductions with only one sequence representative from Arizona. Stacked bars are colored according to location. Lineages were assigned using Pangolin for all sequences uploaded to GISAID as of 4/16/2020. Download FIG S2, PDF file, 0.4 MB. Copyright © 2020 Ladner et al. 2020 Ladner et al. This content is distributed under the terms of the Creative Commons Attribution 4.0 International license.

To estimate when community transmission of SARS-CoV-2 first began in Arizona, we used our Bayesian phylogenetic analysis to estimate dates for the times to most recent common ancestor (TMRCA) of the Arizona genomes within each of these lineages. In total, we sequenced ≥2 viral genomes from 7/12 of the observed lineages (which we name “major lineages”), and TMRCA estimates using these genomes (Fig. 2) suggest that, at the earliest, community transmission in Arizona began around early February 2020. However, we view this as the most extreme estimate because many of these lineages likely represent multiple distinct introductions to Arizona, rather than a single introduction followed by sustained community transmission. For example, the Arizona MRCA for B.1, the inferred oldest lineage containing Arizona genomes, effectively corresponds to the MRCA of the entire B.1 lineage because Arizona genomes assigned to this lineage are scattered throughout this portion of the tree and interspersed with genomes sampled from around the world (Fig. 1). Given the global distribution of this lineage and the high frequency of travel during this time period (15), there have almost certainly been multiple introductions of viruses from this lineage that have led to documented cases in Arizona, but which cannot be distinguished genetically. Therefore, the early February TMRCA likely predates actual community transmission within Arizona. Likewise, the other two lineages with TMRCAs in February (A.1 and B.1.1) both include at least one well-supported sublineage containing a mix of genomes from Arizona and from other locations.

To determine the most likely source of the SARS-CoV-2 introductions to Arizona, we examined the geographic distribution of sequences within each lineage (Fig. 3). For this analysis, we considered the full collection of SARS-CoV-2 genomes available on GISAID (as of 16 April 2020). First, for each of the seven major lineages, Arizona sequences were always sampled after a sequence from that same lineage or sublineage had been sampled elsewhere. Although sequencing efforts can influence this result, it appears unlikely that any of the major lineages emerged in Arizona; rather, they were likely imported. Second, there are very few sequences from Asia clustered within each of the major lineages. Instead, it appears importation was largely driven by domestic travel (in the case of A.1, B.1.2, and B.1.29), or perhaps imported from Europe (B.1 and B.1.1) or Oceania (A.3), although both B.1 and A.3 have also been commonly observed elsewhere in the United States. Given the widespread distribution of many nearly identical genomes, it would be impossible to directly estimate the location of each of these importations beyond the continent level.

The Arizona sequences are largely represented by two lineages, A.1 and B.1, including several sublineages of B.1. We focus the remaining results and discussion on these two lineages. A.1 was identified in Washington State in late February and has since spread across the globe (Fig. 3) (13). It was previously proposed that the A.1 Washington outbreak, announced on 28 February as the second instance of community transmission in the United States (16), is derived from the first Washington case, a lineage A sequence, back in January 2020 (13). For the purposes of our discussion, we only focus on the A.1 sublineage, which, regardless of the ultimate source of A.1, had begun circulating in Washington in the middle of February at the latest.

Eight of the Arizona genomes are members of the A.1 clade, and this includes sequences from at least five counties across Arizona (Table 1; see also Fig. S1). We infer that the MRCA of these Arizona sequences likely existed around 16 February 2020 (95% highest posterior density [HPD], 8 to 24 February). If these eight Arizona genomes stem from a single introduction of the A.1 lineage, this TMRCA estimate suggests the lineage was already present in Arizona prior to when community transmission was announced in Washington, on 29 February (Fig. 2). However, it is likely that the eight A.1 lineage genomes from Arizona arose from multiple introductions into the state, given that the same lineage was being spread throughout the country (15). Although multiple introductions could push the TMRCA estimate of the A.1 introduction to be more recent, we argue that epidemiological data support an initial importation of the A.1 lineage near the dates inferred.

The first A.1 Arizona genome we sequenced was from a sample collected on 5 March from a person who was a household contact of another case, representing the first known community transmission event in Arizona; the other individual tested positive on 3 March and held a health care job in Phoenix (their SARS-CoV-2 genome has not been sequenced) (38). The close epidemiological link between these cases suggests direct transmission of the virus, in which case the first documented example of community transmission in Arizona likely involved an A.1 lineage virus. Given the median incubation time of 5 days (17) and assuming the health care worker was infected by someone traveling directly from Washington with no additional transmission in between, that would place the time of importation to be approximately 28 February. This falls outside our 95% HPD TMRCA estimate of 24 February; however, this timeline makes it clear that even if multiple A.1 introductions caused the TMRCA to be artificially early, it is not by more than a week or two.

**FIG 4:**
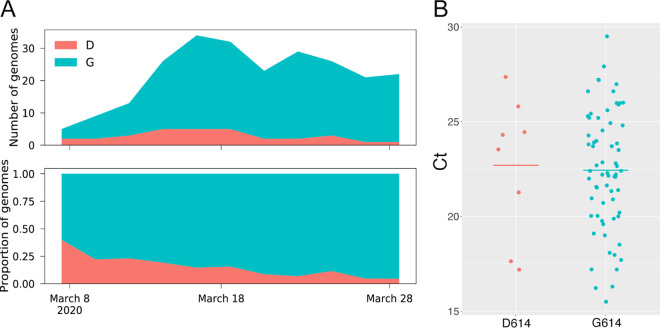
Abundance over time (A) and cycle threshold values (B) for viruses in Arizona with or without the D614G substitution. Both panels were generated using the 79 Arizona genomes we report here. Plots of abundance over time were generated using a window size of 1 week and a step size of 2 days.

Of the 84 Arizona genomes, 72 (85.7%) belong to the B.1 lineage (including the various B.1 sublineages), making B.1 the most abundant lineage in Arizona (as it is globally) (https://github.com/cov-lineages/lineages). Arizona sequences from this lineage were collected from 2 March to 2 April 2020 and were detected in samples from eight counties (Table 1). One of the substitutions that is present in all B.1 lineage viruses occurred in the gene for the spike protein and resulted in an aspartic acid-to-glycine substitution at residue 614 (D614G). Based on viral RNA quantifications from clinical samples, phylogenetic analyses, and *in vitro* experiments (18), it was recently suggested that this substitution may have increased the transmissibility of the virus. Although the outbreak in Arizona was already dominated by B.1 lineage viruses in early March 2020, we did see a gradual increase in the relative proportion of B.1 throughout March and into early April (Fig. 4A). We also compared reverse transcription-PCR (RT-PCR) cycle threshold (*C_T_*) values for clinical samples with and without this substitution. Our results show a trend similar to that reported from patients in Sheffield, England (18), with a lower mean *C_T_* (higher viral load) in samples containing the D614G substitution; however, this is not a statistically significant difference (*P* = 0.85) with our current sample size (Fig. 4B). Combined, these data, along with those published by other groups (18–20), are consistent with a replication and/or transmission advantage of viruses containing the D614G substitution. However, demonstrating a viral mutation has an *in vivo* fitness advantage is difficult and in previous viral outbreaks has contradicted *in vitro* experiments (43, 44). An additional explanation is that the B.1 lineage has simply been increasing in frequency globally, due to chance events that led this lineage to dominate in early outbreaks (e.g., in Italy), from which the virus has been spread widely throughout the world and has seeded outbreaks in many other locations.

The B.1 lineage has also dominated the large outbreak in New York, and in some reports (39), the B.1 lineage has been used as an indication of an epidemiological link to the New York outbreak. However, we would urge caution with this approach. Based on ancestral state reconstructions, this lineage is predicted to have first emerged in Asia or Europe (Nextstrain), and this lineage was observed in multiple European countries, including the large Italian outbreak, before it was first documented in New York (Nextstrain). In fact, just within Arizona, we have documented at least two instances in which B.1 lineage viruses were imported directly from Europe. The second case of COVID-19 in Arizona was reported on 3 March from a traveler who returned from France on 27 February (40). This individual self-reported being symptomatic on the plane back to Phoenix and went to multiple social gatherings before being officially diagnosed (41). At one of these social events, at least one other individual was infected (42). Virus from this individual was sequenced by the CDC (EPI_ISL_420784), and the genome belongs to the B.1 lineage (sublineage B.1.1; see Fig. S1). We also have records shared to us by the Coconino Health and Human Services Department that one of the B.1 genomes we sequenced from Coconino County came from an individual who had recently traveled to Rome, Italy, and who presumably was infected there. Thus, although it is tempting to speculate that most of the B.1 infections across the United States came from the New York outbreak, we show at least two confirmed instances of direct importation of B.1 into Arizona from Europe.

### Nonsynonymous mutations of interest observed in Arizona SARS-CoV-2 sequences.

**FIG 5:**
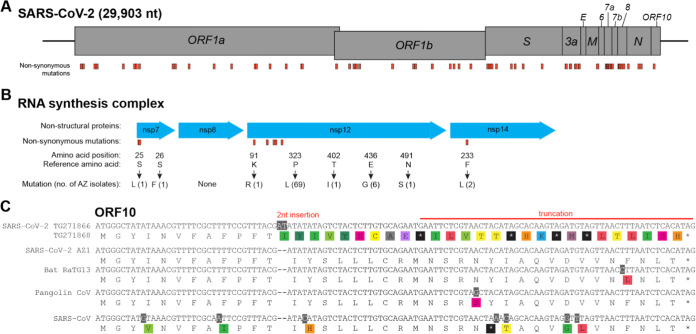
Nonsynonymous mutations in Arizona isolates. (A) Diagram showing the SARS-CoV-2 genome and annotated open reading frames. The genome positions of nonsynonymous mutations in Arizona SARS-CoV-2 isolates are indicated in orange. (B) Nonsynonymous mutations of Arizona isolates in nsp’s involved in the SARS-CoV-2 RNA synthesis complex. Mutations (indicated in orange) are labeled by amino acid position within the protein, reference amino acid, amino acid change, and number of Arizona isolates with the mutation. (C) ORF10 alignment showing a 2-nucleotide insertion and subsequent early truncation in two Arizona SARS-CoV-2 isolates. GenBank and GISAID accession numbers: SARS-CoV-2 AZ-TG271866 (EPI_ISL_427271), SARS-CoV-2 AZ-TG271868 (EPI_ISL_427272), SARS-CoV-2 AZ1 (MN997409.1, EPI_ISL_406223), Bat-RaTG13 (MN996532.1), Pangolin (EPI_ISL_410721), and SARS-CoV (NC_004718.3).

Like all RNA viruses, SARS-CoV-2 accumulates mutations over time, some of which may impact virulence, replication, and intervention strategies and some of which have no functional, clinical, or antigenic importance. We identified nonsynonymous mutations in coding sequences of SARS-CoV-2 genomes from Arizona (Fig. 5; see also Table S1). These include mutations in the spike protein, nonstructural proteins (nsps) involved in RNA synthesis, nucleocapsid protein, and the putative ORF10. Several of these mutations have also been reported as being associated with SARS-CoV-2 sequences from Europe (22). Below, we hypothesize about potential phenotypic impacts of these substitutions; however, it is important to note that experimental studies need to be conducted to test these hypotheses, and the vast majority of substitutions that occur during viral replication will not have a significant impact on virulence or transmissibility.

As the virus genome mutates there are several concerns, such as evasion of therapeutics and vaccines but also regarding diagnostics. In order to identify any mutations that could affect the specificity of currently used RT-PCR assays, an *in silico* approach was employed. A screen of 12 current primer/probe sets (Table S4) demonstrates that seven widely used assays are valid in this isolate set, yielding no predicted false negatives based on exact primer/probe matches. Several of the primers/probes align with stretches of “N” characters in individual genomes and the amplification potential is therefore ambiguous. Interestingly, the CDC nCoV_N1 assay demonstrates mismatches in six of the genomes screened in this study, including one genome from Arizona (TG271862, Cochise County). This is not a guarantee of future validity; however, automated *in silico* methods offer nearly effortless monitoring.

10.1128/mBio.02107-20.7 TABLE S4 *In silico* screen of commonly used primers/probes to detect SARS-CoV-2. Download Table S4, PDF file, 0.1 MB. Copyright © 2020 Ladner et al. 2020 Ladner et al. This content is distributed under the terms of the Creative Commons Attribution 4.0 International license.

### Spike.

The SARS-CoV-2 spike (S) protein mediates receptor binding and cell entry and is the primary target of neutralizing antibodies (23). Mutations in the spike protein may have implications for viral entry and recognition by the immune system. We found several mutations in the spike protein gene, including in two related isolates (TGEN-CoV-AZ-WMTS-TG268282 and TGEN-CoV-AZ-WMTS-TG271435) harboring an alanine to valine substitution at Spike amino acid residue 475 (A475V, nucleotide position 22986) in the receptor-binding domain (RBD). Structural studies indicate that A475 interacts with S19 of ACE2 (24, 25).

### Nsp12 and RNA synthesis complex.

During replication of the SARS-CoV-2 RNA genome, RNA synthesis is driven by the key component nsp12 RNA-dependent RNA polymerase in complex with nsp7 and nsp8 (26, 27). Studies of SARS-CoV replication demonstrate that nsp14 regulates replication fidelity through its 3′-to-5′ exonuclease activity (28, 29). Mutations in nsp7, nsp8, nsp12, and nsp14 may therefore affect viral RNA synthesis and susceptibility to antiviral treatments such as remdesivir (30). We identified several nonsynonymous mutations in nsp7 residue 25 (S→L) and 26 (S→F), but none in nsp8 (Fig. 5B). One of the nsp12 mutations at residue 323 (*P*→L) identified in 69 Arizona sequences was previously associated with SARS-CoV-2 sequences from Europe (22). We did not find nsp12 mutations at sites predicted to be the contact interface with remdesivir (30). Finally, the two related isolates with nsp14 mutation at residue 233 (F→L) were the same genomes harboring the spike RBD A475V mutation.

### Nucleocapsid.

The nucleocapsid (N) protein encapsulates the genomic RNA and is a target for diagnostic and therapeutic applications (31). The N protein is also associated with replication-transcription complexes and facilitates template switching during viral subgenomic mRNA synthesis (32). N is expressed at high levels during early stages of replication and, like the S protein, is also a major immunogenic target of antibodies (33). Five AZ sequences had a triple nucleotide substitution (GGG→AAC) that resulted in a tandem amino acid change in the N protein at residues 203 and 204 (RG→KR). Over the relatively short time frame of SARS-CoV-2 evolution, these tandem substitutions are relatively uncommon (22).

### ORF10.

ORF10 is a short putative protein of unknown function, predicted in the 3′ end of the SARS-CoV-2 genome, which is conserved in the closely related bat and pangolin coronavirus sequences (Fig. 5C). We identified a 2-nucleotide insertion in ORF10 within two AZ genomes that results in a premature stop codon and early truncation of ORF10. A similar truncation is present in the SARS-CoV genome due to the presence of an upstream stop codon. This may indicate a region of the virus genome with relaxed evolutionary constraints, consistent with a report that did not detect subgenomic ORF10 mRNA *in vitro* (34).

### Conclusions.

Based on our phylogenetic analysis, it is clear that the ongoing COVID-19 outbreak in Arizona has been fueled by multiple distinct introductions of SARS-CoV-2 to the state. We estimate a minimum of 11 introductions over the course of February and March, though this is surely an underestimate. By estimating the timing of introductions, we find no evidence for cryptic community transmission in Arizona prior to late January. Rather, our analyses indicate that community transmission likely did not occur within Arizona until at the earliest early- to mid-February, when viruses from lineages B.1 and A.1 may have been first introduced. It appears that most of the introductions of SARS-CoV-2 to Arizona have had domestic origins, in line with reports from other states (15); however, there have also been instances of Arizona cases linked directly to international travel, and these have likely also contributed to the local outbreak. Several nonsynonymous mutations were identified in the Arizona isolates, including within regions of the receptor-binding domain of the spike protein and nonstructural proteins involved in the RNA synthesis complex. However, we see very little evidence for mutations that will impact the most commonly used molecular diagnostics. The functional consequences of the observed mutations are unknown, highlighting the need for mechanistic studies. Our phylodynamic tracing provides unique epidemiological insights into the origins and transmission of SARS-CoV-2 in Arizona and will form the basis for future understanding as the pandemic continues.

## MATERIALS AND METHODS

### Reference genome.

Any sequence positions mentioned in this work refer to GenBank sequence NC_045512.2, a genome isolated and sequenced from Wuhan, China, early in the pandemic.

### TGen North genome sequencing.

RNA was extracted from specimen transport medium with a quick viral RNA kit (Zymo Research). Total RNA sequencing libraries were prepared with the SMART-Seq stranded kit (TaKaRa) or the Ovation RNA-Seq system (NuGEN). Libraries were sequenced on a NextSeq (high-output kit; Illumina). Viral genome consensus sequences for each sample were constructed using TGen’s amplicon sequencing analysis pipeline (ASAP; https://github.com/TGenNorth/ASAP), which consists of mapping the reads to a reference genome (hCoV-19/USA/AZ1/2020|EPI_ISL_406223 (https://www.gisaid.org/) and analyzing the alignment pileup position by position to determine coverage depth, coverage breadth, and the consensus sequence. Positions covered by fewer than 10 reads were considered a gap in coverage and were converted to Ns. Consensus sequences were saved and used for further analysis when they had >90% breadth of coverage and >30× average depth of coverage.

### University of Arizona genome sequencing.

After sample collection, the nasopharyngeal swab was soaked in TRIzol (Thermo Fisher) and removed. Total RNA was then extracted from 400 μl of TRIzol with a Direct-zol RNA isolation kit (Zymo Research) according to the manufacturer’s instructions. RNA was eluted in 30 μl of nuclease-free water.

We used primers and methods from the ARTIC consortium (https://artic.network/) with the following modifications. cDNA synthesis was performed with GOscript (Promega**)** using 10 μl of RNA in a final volume of 20 μl according to the manufacturer’s instructions. Next, a multiplex PCR amplifying overlapping 400-bp amplicons was performed with the V2 set of primers designed by the ARTIC group. We used 2.5 μl of cDNA in each 25-μl reaction with the Q5 Hot Start high-fidelity DNA polymerase (NEB), with two separate reaction mixtures containing each of the nonoverlapping primer pools. We used an initial denaturation step of 98°C for 30 s and then 35 cycles at 98°C for 10 s and 65°C for 2 min.

The reaction mixtures were pooled and visualized on a 2% agarose gel to confirm successful amplification. Amplicons were then cleaned with a 1:1 mixture of AMPure XP magnetic beads (Beckman Coulter). The mixture was incubated for 5 min at room temperature and then placed on a magnetic rack until all of the beads were pulled out of the solution. The remaining liquid was pulled off and discarded. Next, 200 μl of 80% ethanol was used twice to wash the bead mixture. The beads were allowed to dry and resuspended in 30 μl of water. After a 5-min incubation, the tube was placed back on a magnetic rack until the beads were pulled out of the solution. Then, a 30-μl portion of the cleaned amplicons was transferred to a fresh tube.

The remainder of the protocol was identical to the ARTIC protocol which includes end-repair, ligation of Oxford Nanopore sequencing adapters, and additional cleaning steps using the AMPure XP beads. The final prepared library was loaded onto a flongle inserted into a MinION sequencer. Sequence data were collected for 12 h. In order to remove the sequencing adapter and primer sequence, we trimmed the first 40 bp off the reads. Reads were then aligned to a SARS-CoV-2 reference sequence (MN908947) using Geneious Prime (Biomatters, Inc.). A consensus sequence was generated from these reads for sections that contained >40× coverage with Ns placed at sites with lower coverage.

### Arizona State University genome sequencing.

SARS-CoV-2 genomes were sequenced from nasopharyngeal swabs as previously described (35). Briefly, total nucleic acid was extracted using the bioMérieux eMAG platform. RNA was subjected to Ribo-Zero Gold depletion, TruSeq RNA library preparation, and sequenced on Illumina NextSeq (2 × 76). Sequencing reads were quality filtered with BBtools (BBMap; Bushnell B.; https://sourceforge.net/projects/bbmap/) and mapped to a SARS-CoV-2 reference genome (MN908947).

### Bioinformatics.

Sequences included in the analyses presented here were derived from GISAID (accessed on 16 April 2020), NCBI GenBank, and sequences generated by our teams at Northern Arizona University and TGen North (*n* = 75), Arizona State University (*n* = 3), and University of Arizona (*n* = 1) (collectively referred to as the “Arizona sequences”). To support efficient Bayesian phylogenetic analysis of this large number of sequences, we developed genome-sampler (36), a novel protocol and software for sampling sequences from GISAID across time of sequence acquisition, geographic source of sequence, and SARS-CoV-2 diversity. The software developed for this workflow is available at https://github.com/caporaso-lab/az-covid-1, and our application of this workflow is detailed in our protocol in the supplemental methods (see Text S1 in the supplemental material).

10.1128/mBio.02107-20.1 TEXT S1 Supplemental methods. Download Text S1, PDF file, 0.1 MB. Copyright © 2020 Ladner et al. 2020 Ladner et al. This content is distributed under the terms of the Creative Commons Attribution 4.0 International license.

10.1128/mBio.02107-20.8 TABLE S5 Model comparison results from the generalized stepping-stone sampling analyses. The best-fitting model likelihood and estimates are indicated in boldface. Download Table S5, PDF file, 0.1 MB. Copyright © 2020 Ladner et al. 2020 Ladner et al. This content is distributed under the terms of the Creative Commons Attribution 4.0 International license.
