# Supplementary material for: An Early Pandemic Analysis of SARS-CoV-2 Population Structure and Dynamics in Arizona
Source: mBio. 2020 Sep 4;11(5):e02107-20. doi: 10.1128/mBio.02107-20 (PMC7474171; doi:10.1128/mBio.02107-20)
Supplement: TABLE S3 [file mBio.02107-20-st003.pdf]

Table S3. Genome representation from each Arizona county. This does not include all genomes because some are from unknown counties.

**County**	**Number of positive cases***	**Number of genomes sequenced**
Apache	118	0
Cochise	18	1
Coconino	299	22
Gila	5	0
Graham	2	1
Greenlee	2	0
La Paz	5	1
Maricopa	2,264	31
Mohave	51	1
Navajo	410	4
Pima	760	4
Pinal	197	7
Santa Cruz	14	0
Yavapai	68	3
Yuma	21	1
**Totals**	**4234**	**76**
* As of 4/16/2020		
