# Supplementary material for: An Early Pandemic Analysis of SARS-CoV-2 Population Structure and Dynamics in Arizona
Source: mBio. 2020 Sep 4;11(5):e02107-20. doi: 10.1128/mBio.02107-20 (PMC7474171; doi:10.1128/mBio.02107-20)
Supplement: FIG S1 [file mBio.02107-20-sf001.pdf]

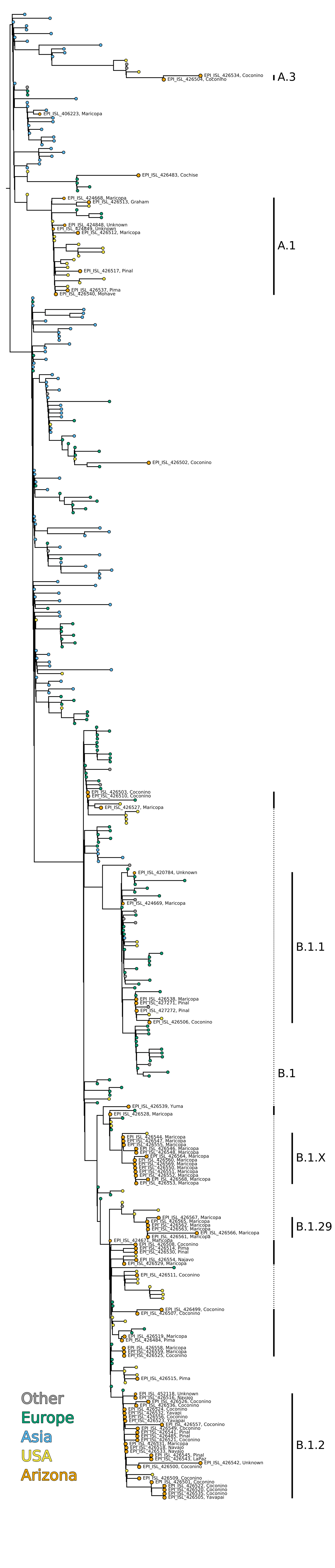

EPI_ISL_426534, Coconino
EPI_ISL_426504, Coconino
A.3
EPI_ISL_406223, Maricopa
EPI_ISL_426483, Cochise
EPI_ISL_424668, Maricopa
EPI_ISL_426513, Graham
EPI_ISL_424848, Unknown
EPI_ISL_424849, Unknown
EPI_ISL_426512, Maricopa
A.1
EPI_ISL_426517, Pinal
EPI_ISL_426537, Pima
EPI_ISL_426540, Mohave
EPI_ISL_426502, Coconino
EPI_ISL_426503, Coconino
EPI_ISL_426510, Coconino
EPI_ISL_426527, Maricopa
EPI_ISL_420784, Unknown
EPI_ISL_424669, Maricopa
B.1.1
EPI_ISL_426538, Maricopa
EPI_ISL_427271, Pinal
EPI_ISL_427272, Pinal
EPI_ISL_426506, Coconino
B.1
EPI_ISL_426539, Yuma
EPI_ISL_426528, Maricopa
EPI_ISL_426544, Maricopa
EPI_ISL_426547, Maricopa
EPI_ISL_426555, Maricopa
EPI_ISL_426546, Maricopa
EPI_ISL_426548, Maricopa
EPI_ISL_426564, Maricopa
EPI_ISL_426560, Maricopa
EPI_ISL_426569, Maricopa
EPI_ISL_426550, Maricopa
EPI_ISL_426551, Maricopa
EPI_ISL_426552, Maricopa
EPI_ISL_426568, Maricopa
EPI_ISL_426553, Maricopa
B.1.X
EPI_ISL_426567, Maricopa
EPI_ISL_426565, Maricopa
EPI_ISL_426562, Maricopa
EPI_ISL_426563, Maricopa
EPI_ISL_426566, Maricopa
EPI_ISL_426561, Maricopa
B.1.29
EPI_ISL_424671, Maricopa
EPI_ISL_426508, Coconino
EPI_ISL_426514, Pima
EPI_ISL_426530, Pinal
EPI_ISL_426554, Najavo
EPI_ISL_426529, Maricopa
EPI_ISL_426511, Coconino
EPI_ISL_426499, Coconino
EPI_ISL_426507, Coconino
EPI_ISL_426519, Maricopa
EPI_ISL_426484, Pima
EPI_ISL_426558, Maricopa
EPI_ISL_426559, Maricopa
EPI_ISL_426525, Coconino
EPI_ISL_426515, Pima
EPI_ISL_452118, Unknown
EPI_ISL_426516, Navajo
EPI_ISL_426526, Coconino
EPI_ISL_426536, Coconino
EPI_ISL_426524, Coconino
EPI_ISL_426532, Yavapi
EPI_ISL_426556, Coconino
EPI_ISL_426523, Yavapai
EPI_ISL_426557, Coconino
EPI_ISL_426549, Coconino
EPI_ISL_426541, Pinal
EPI_ISL_426485, Pinal
EPI_ISL_426521, Coconino
EPI_ISL_426531, Maricopa
EPI_ISL_426518, Navajo
EPI_ISL_426533, Navajo
EPI_ISL_426545, Pinal
EPI_ISL_426543, LaPaz
EPI_ISL_426542, Unknown
EPI_ISL_426500, Coconino
EPI_ISL_426509, Coconino
EPI_ISL_426501, Coconino
EPI_ISL_426522, Coconino
EPI_ISL_426520, Coconino
EPI_ISL_426535, Coconino
EPI_ISL_426505, Yavapai
B.1.2
Other
Europe
Asia
USA
Arizona

Figure S1. Maximum-likelihood phylogeny inferred from 388 SARS-CoV-2 genomes, including 84 from Arizona and 304 representatives from around the world. Tips are colored by origin of sequence, and major lineages assigned by Pangolin, with more than 2 sequence representatives from Arizona, are labeled and indicated by black vertical bars (Note: vertical bars do not necessarily cover all genomes from a given Pangolin lineage, only those from Arizona). B.1.X is a well-supported sublineage of B.1 that has not been named by Pangolin. Genomes from Arizona are labeled with the associated GISAID accession number and Arizona County of origin, when available. The tree was visualized with a custom Python script that utilized the software package BALTIC (https://github.com/evogytis/baltic).
