# Supplementary material for: An Early Pandemic Analysis of SARS-CoV-2 Population Structure and Dynamics in Arizona
Source: mBio. 2020 Sep 4;11(5):e02107-20. doi: 10.1128/mBio.02107-20 (PMC7474171; doi:10.1128/mBio.02107-20)
Supplement: FIG S2 [file mBio.02107-20-sf002.pdf]

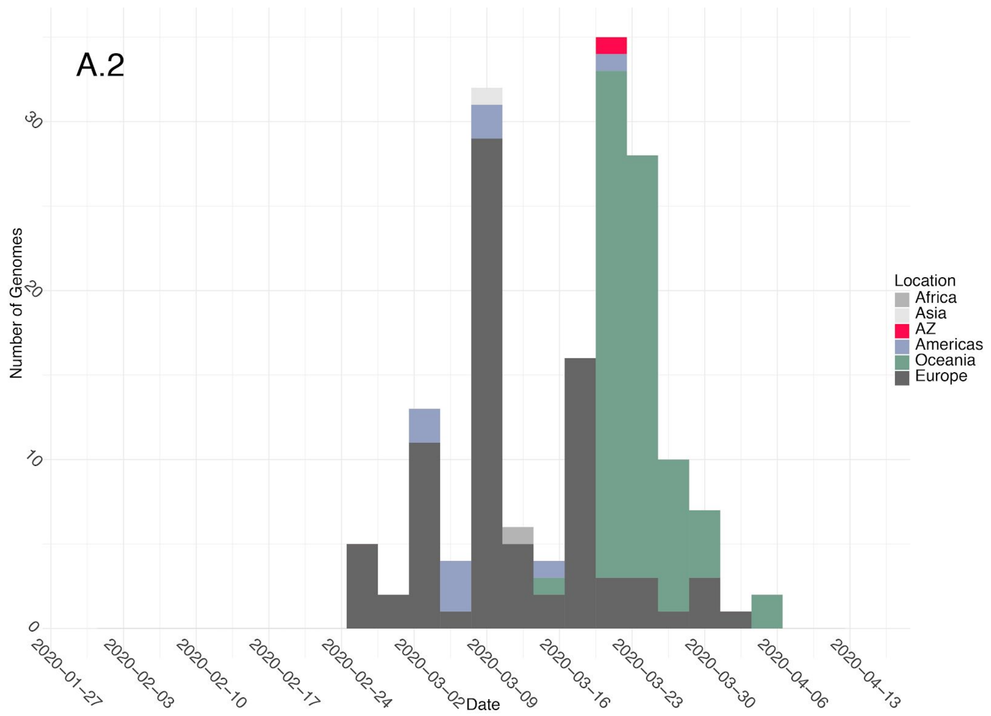
A.2
Number of Genomes
0
10
20
30
2020–01–27
2020–02–03
2020–02–10
2020–02–17
2020–02–24
2020–03–02
2020–03–09
2020–03–16
2020–03–23
2020–03–30
2020–04–06
2020–04–13
Date
Location
Africa
Asia
AZ
Americas
Oceania
Europe

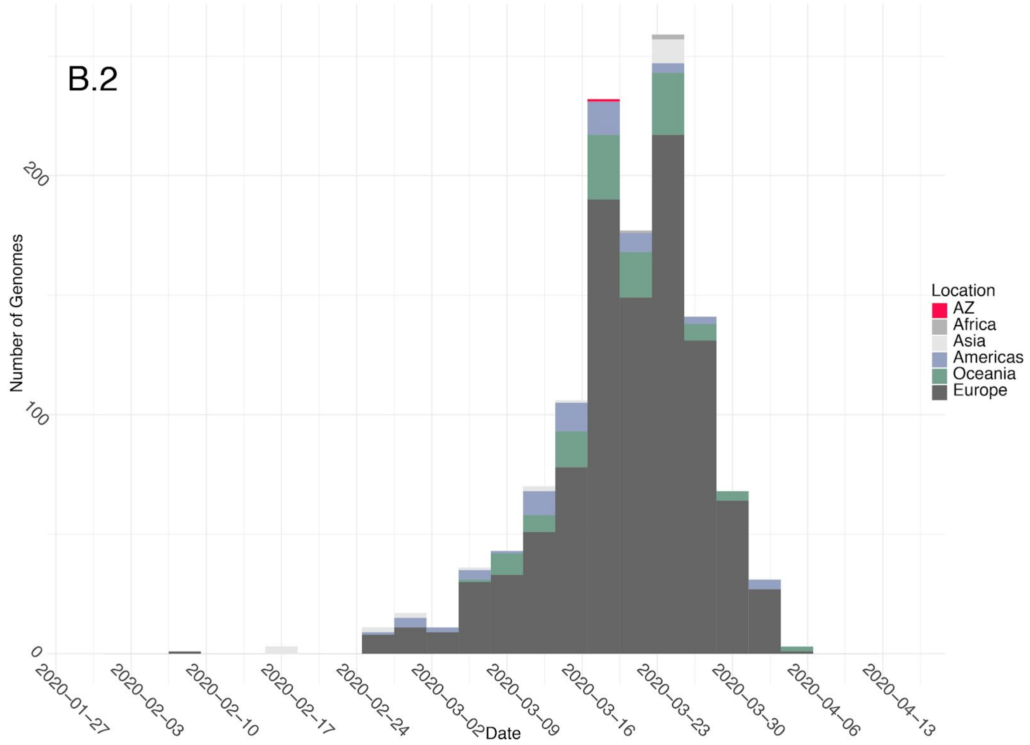
B.2
Number of Genomes
0
100
200
2020–01–27
2020–02–03
2020–02–10
2020–02–17
2020–02–24
2020–03–02
2020–03–09
2020–03–16
2020–03–23
2020–03–30
2020–04–06
2020–04–13
Date
Location
AZ
Africa
Asia
Americas
Oceania
Europe

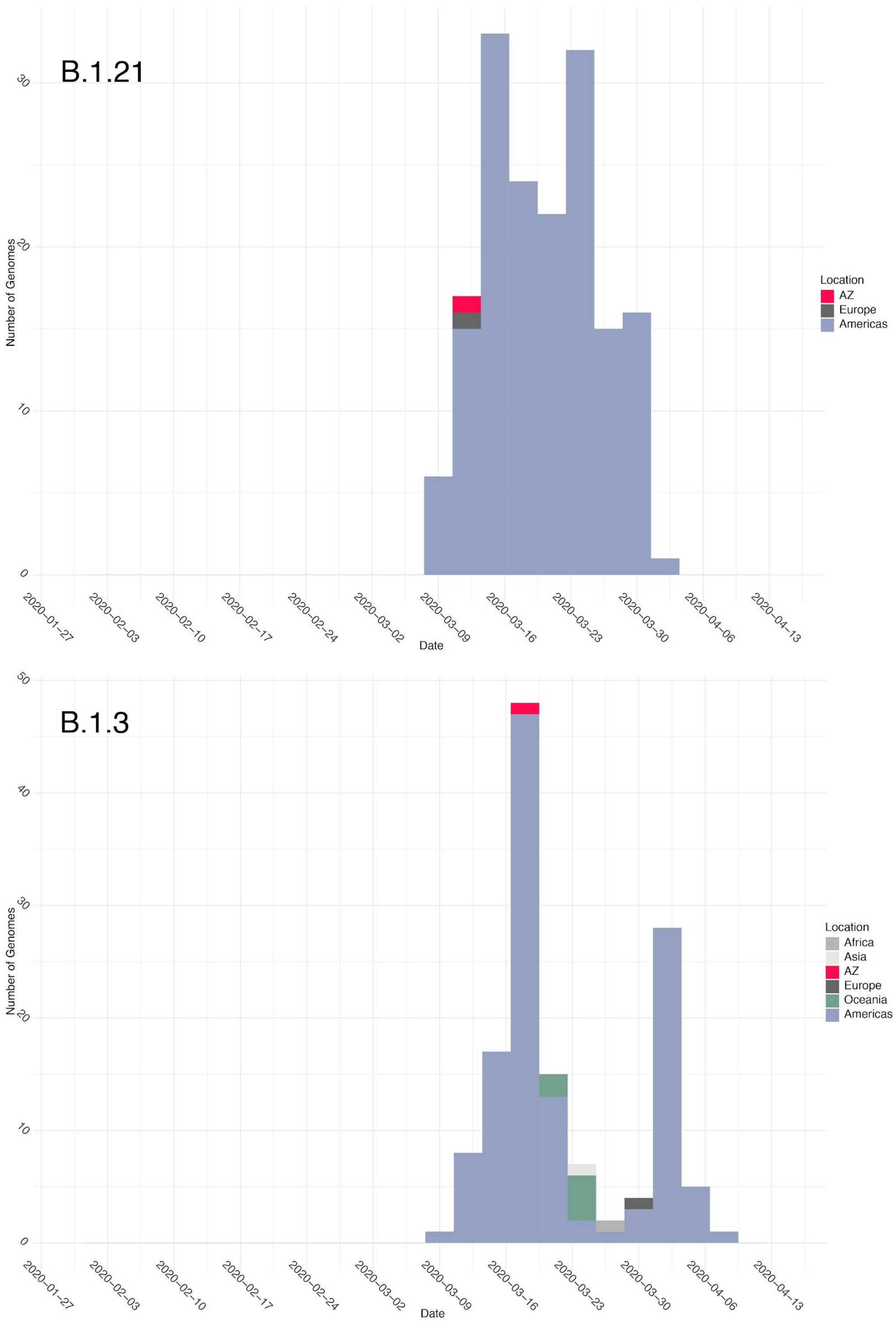

Figure S2. Sequence database representation through time for the 4 introductions with only 1 sequence representative from Arizona. Stacked bars are colored according to location. Lineages were assigned using Pangolin for all sequences uploaded to GISAID as of 4/16/2020.
