# Supplementary material for: An Early Pandemic Analysis of SARS-CoV-2 Population Structure and Dynamics in Arizona
Source: mBio. 2020 Sep 4;11(5):e02107-20. doi: 10.1128/mBio.02107-20 (PMC7474171; doi:10.1128/mBio.02107-20)
Supplement: TABLE S4 [file mBio.02107-20-st004.pdf]

Table S4. *In silico* screen of commonly used primers/probes to detect SARS-CoV-2.

Assay	Forward	Reverse	Probe	Total	Hits	Misses	Ambiguous
Orf1ab	CCCTGTGGGTTTTACACTTAA	ACGATTGTGCATCAGCTGA	CCGTCTGCGGTATGTGGAAAGGTTATGG	388	370	0	18
N (China)	GGGGAACTTCTCCTGCTAGAAT	CAGACATTTTGCTCTCAAGCTG	TTGCTGCTGCTTGACAGATT	388	327	58	3
n_cov_n1	GACCCCAAAATCAGCGAAAT	TCTGGTTACTGCCAGTTGAAT	ACCCCGCATTACGTTTGGTGGACC	388	379	6	3
n_cov_n2	TTACAAACATTGGCCGCAAA	GCGCGACATTCCGAAGAA	ACAATTTGCCCCCAGCGCTTCAG	388	386	0	2
n_cov_n3	GGGAGCCTTGAATACACCAAAA	TGTAGCACGATTGCAGCATTG	AYCACATTGGCACCCGCAATCCTG	388	386	0	2
E (Sarbeco)	ACAGGTACGTTAATAGTTAATAGCGT	ATATTGCAGCAGTACGCACACA	ACACTAGCCATCCTTACTGCGCTTCG	388	384	2	2
nsp10 (China)	CCCTGTGGGTTTTACACTTAA	ACGATTGTGCATCAGCTGA	CCGTCTGCGGTATGTGGAAAGGTTATGG	388	370	0	18
nsp14	TAATCAGACAAGGAACTGATTA	CGAAGGTGTGACTTCCATG	GCAAATTGTGCAATTTGCGG	388	384	2	2
N (HKU)	TAATCAGACAAGGAACTGATTA	CGAAGGTGTGACTTCCATG	GCAAATTGTGCAATTTGCGG	388	383	2	3
nsp14 (HKU)	TGGGG**Y**TTTACRGGTAACCT	AAC**R**CGCTTAACAAAGCACTC	TAGTTGTGATGC**W**ATCATGACTAG	388	385	0	3
RdRp (IP2)	ATGAGCTTAGTCCTGTTG	CTCCCTTTGTTGTGTTGT	AGATGTCTTGTGCTGCCGGTA	388	387	0	1
RDRP (IP4)	GGTAACTGGTATGATTTCG	CTGGTCAAGGTTAATATAGG	TCATACAAACCACGCCAGG	388	388	0	0
