# Supplementary material for: An Early Pandemic Analysis of SARS-CoV-2 Population Structure and Dynamics in Arizona
Source: mBio. 2020 Sep 4;11(5):e02107-20. doi: 10.1128/mBio.02107-20 (PMC7474171; doi:10.1128/mBio.02107-20)
Supplement: TEXT S1 [file mBio.02107-20-s0001.pdf]

Supplementary Methods

Genome Sampler protocol

This protocol works as follows:

1. Download SARS-CoV-2 sequences from GISAID (9230 at time of download)
2. Clean-up and filter GISAID sequences (8659 sequences remained after these filters)
    a. Specific steps were:
        i. Filter sequences that contain spaces (spaces are non-IUPAC characters(37)) - this resulted in 408 sequences being discarded.
        ii. Remove any gap ("-") characters (sequences are unaligned, so should not contain gaps)
        iii. Filter sequences that are composed of >10% N characters - this resulted in 163 sequences being discarded.
        iv. Replace all spaces in sequence header lines with underscores
3. Compile initial sequence collection containing Arizona sequences, reference sequence from NCBI GenBank (NC_045512.2),seven randomly sampled sequences per week from GISAID, WA1 (MN985325.1), and AZ1 (MN997409).
4. We then searched all of GISAID against the sequence collection compiled in Step 3 using vsearch's usearch_global option, and identified sequences that most closely matched the Arizona sequences, to ensure that we were including the closest relatives of Arizona sequences to contextualize clades containing Arizona sequences (1). The collection of best hits was sampled to result in a geographically dispersed collection of sequence sources. Those sequences were added to the sequence collection from Step 3, and ensure that monophylies of Arizona sequences are not artifacts of our sequence sampling approach.
5. All GISAID sequences that were not clustered by the previous step were clustered with vsearch's cluster_fast option at 99.9% percent identity. Cluster centroid sequences were added to the sequence collection generated in step 4. This ensured that a divergent collection of the SARS-CoV-2 genomes was represented in our data set.
6. An initial alignment was constructed with MAFFT followed by the construction of a neighbor joining tree. Several sequences that were suspected of being low quality at this stage, including both GISAID and Arizona sequences, were removed from the sequence collection.

The sequences resulting from this workflow were used as the starting point for all downstream analyses.

Phylogenetics

Maximum-likelihood phylogenies were generated using RAxML-NG v0.5.1b (2) with the GTR+G4 model, as indicated by a substitution model selection analysis carried out in IQTree, with 20 distinct starting trees and 100 bootstrap replicates. Lineage naming for all Arizona sequences was performed with the software Pangolin, which is a real time dynamic tool for

assigning lineages to SARS-CoV-2 sequences based on shared mutations and phylogenetic support (3). Pangolin was run twice (May 3rd and July 16th, 2020) and the most specific lineage name for each sequence was used.

We employed a Bayesian molecular clock method implemented in the BEAST v1.10.5 (4) software package to estimate divergence times for the total SARS-CoV-2 dataset as well as several Arizona-specific lineages, and overall evolutionary rates. To determine the best fitting clock and demographic model combinations for these data, the generalized stepping stone marginal likelihood estimator (5) was employed to compare the Strict or Uncorrelated Lognormal (UCLN) (6) clock models combined with Exponential or Bayesian Skygrid demographic (7) models. The four model combinations were each iterated for 100,000,000 generations, where Markov chains were sampled every 10,000 generations. We found that the Strict clock and Bayesian Skygrid combination (Strict Skygrid) outperformed the other three combinations (Supplementary Table 5). An additional three chains using the Strict Skygrid model combination were run for 100,000,000 generations and sampled every 10,000 generations. We found convergence within and among chains using Tracer v1.6. LogCombiner was used to merge the four different chains of each model combination, after discarding the first 10% as burn in (10,000,000 generations per chain), and then resampling every 30,000 generations. The resulting file was input to TreeAnnotator to produce maximum clade credibility trees with median height estimates. BALTIC (https://github.com/evogytis/baltic) was used for visualizing trees and for parsing TMRCAs from the trees sampled during the BEAST analysis. Monophyly was not enforced for the different AZ genomes within each lineage. Therefore, the clades defined by these MRCAs may include non-AZ sequences and the non-AZ members of these clades are likely to vary among different sampled trees. We consider these TMRCAs to provide conservative estimates for how early SARS-CoV-2 began circulating locally in AZ.

In silico **PCR screen**

The *in silico* PCR screen was performed with an in silico PCR script (https://github.com/TGenNorth/vipr) using published primers and probes (Supplementary Table 4). Positives were identified by exact nucleotide matches of both primers and probe. Mishits were manually identified through visual inspection of the primer/probe alignments. If a primer/probe aligned against a genome with an “N” character, the hit was considered as ambiguous. The Arizona SARS-CoV-2 isolates were aligned against reference genome AZ1 (GenBank accession MN997409, GISAID accession EPI_ISL_406223) and non-synonymous variants in the coding sequences were called by Geneious Prime (2020.0.5).

References

1. Rognes T, Flouri T, Nichols B, Quince C, Mahé F. 2016. VSEARCH: a versatile open source tool for metagenomics. PeerJ 4:e2584.

2. Kozlov AM, Darriba D, Flouri T, Morel B, Stamatakis A. 2019. RAxML-NG: a fast, scalable and user-friendly tool for maximum likelihood phylogenetic inference. Bioinformatics 35:4453–4455.

3. Rambaut A, Holmes EC, O'Toole Á, Hill V, McCrone JT, Ruis C, du Plessis L, Pybus OG. 2020. A dynamic nomenclature proposal for SARS-CoV-2 lineages to assist genomic epidemiology. Nat Microbiol 164:2417.

4. Suchard MA, Lemey P, Baele G, Ayres DL, Drummond AJ, Rambaut A. 2018. Bayesian phylogenetic and phylodynamic data integration using BEAST 1.10. Virus Evol 4:vey016.

5. Baele G, Lemey P, Bedford T, Rambaut A, Suchard MA, Alekseyenko AV. 2012. Improving the accuracy of demographic and molecular clock model comparison while accommodating phylogenetic uncertainty. Mol Biol Evol 29:2157–2167.

6. Drummond AJ, Ho SYW, Phillips MJ, Rambaut A. 2006. Relaxed phylogenetics and dating with confidence. PLoS Biol 4:e88.

7. Gill MS, Lemey P, Faria NR, Rambaut A, Shapiro B, Suchard MA. 2013. Improving Bayesian population dynamics inference: a coalescent-based model for multiple loci. Mol Biol Evol 30:713–724.
