# Supplementary material for: An Early Pandemic Analysis of SARS-CoV-2 Population Structure and Dynamics in Arizona
Source: mBio. 2020 Sep 4;11(5):e02107-20. doi: 10.1128/mBio.02107-20 (PMC7474171; doi:10.1128/mBio.02107-20)
Supplement: TABLE S5 [file mBio.02107-20-st005.pdf]

Table S5. Model comparison results from the generalized stepping stone sampling analyses. The best-fitting model likelihood and estimates are bolded.

Model Combination	log marginal likelihood	Mean TMRCA Estimate	Median TMRCA Estimate	TMRCA 95% CI	Dates 95% CI	meanRate
Strict Exponential	-46733	2019.8321	2019.837	2019.7464-2019.9111	Sept. 30-Nov. 29, 2019	1.01E-03
**Strict Skygrid**	**-46678**	**2019.8968**	**2019.904**	**2019.8228-2019.9578**	**Oct. 28-Dec. 16, 2019**	**9.74E-04**
UCLN Exponential	-46734	2019.8225	2019.8332	2019.7024-2019.9207	Sept. 14-Dec. 3, 2019	1.03E-03
UCLN Skygrid	-46695	2019.9183	2019.9274	2019.8413-2019.9772	Nov. 11-Dec. 23, 2019	1.01E-03
